# Reprogramming human A375 amelanotic melanoma cells by catalase overexpression: Reversion or promotion of malignancy by inducing melanogenesis or metastasis

**DOI:** 10.18632/oncotarget.9220

**Published:** 2016-05-07

**Authors:** Candelaria Bracalente, Noelia Salguero, Cintia Notcovich, Carolina B. Müller, Leonardo L. da Motta, Fabio Klamt, Irene L. Ibañez, Hebe Durán

**Affiliations:** ^1^ Departamento de Micro y Nanotecnología, Comisión Nacional de Energía Atómica, San Martín, Buenos Aires, B1650KNA, Argentina; ^2^ Consejo Nacional de Investigaciones Científicas y Tecnológicas, Buenos Aires, C1033AAJ, Argentina; ^3^ Laboratório de Bioquímica Celular, Departamento de Bioquímica, Instituto de Ciências Básicas da Saúde, Universidade Federal do Rio Grande do Sul, Porto Alegre, 90035 003, Brasil; ^4^ Escuela de Ciencia y Tecnología, Universidad Nacional de San Martín, San Martín, Buenos Aires, B1650HMP, Argentina

**Keywords:** melanoma, catalase, ROS, melanogenesis and metastasis

## Abstract

Advanced melanoma is the most aggressive form of skin cancer. It is highly metastatic and dysfunctional in melanogenesis; two processes that are induced by H_2_O_2_. This work presents a melanoma cell model with low levels of H_2_O_2_ induced by catalase overexpression to study differentiation/dedifferentiation processes. Three clones (A7, C10 and G10) of human A375 amelanotic melanoma cells with quite distinct phenotypes were obtained. These clones faced H_2_O_2_ scavenging by two main strategies. One developed by clone G10 where ROS increased. This resulted in G10 migration and metastasis associated with the increased of cofilin-1 and CAP1. The other strategy was observed in clone A7 and C10, where ROS levels were maintained reversing malignant features. Particularly, C10 was not tumorigenic, while A7 reversed the amelanotic phenotype by increasing melanin content and melanocytic differentiation markers. These clones allowed the study of potential differentiation and migration markers and its association with ROS levels *in vitro* and *in vivo*, providing a new melanoma model with different degree of malignancy.

## INTRODUCTION

Melanoma arises from malignant transformation of melanocytes. Besides, it is highly metastatic and among the most resistant cancers to treatments [1]. Loss of pigmentation in melanoma is common in advanced lesions because of dysfunction in melanogenesis proteins. Tyrosinase (TYR) and the tyrosinase related proteins 1 and 2 (TYRP1 and TYRP2 respectively), are the main proteins involved in melanogenesis [2].

Melanoma cells produce high amounts of reactive oxygen species (ROS) compared with their non-tumoral counterpart [3, 4]. ROS participate in tumor development by inducing oncogenic mutation and signal transduction pathways associated with cell proliferation [5, 6], differentiation [7] and the pro-invasive metastatic program [7–12]. Antioxidant enzymes, such as superoxide dismutase (SOD), catalase, peroxidases and peroxiredoxins, control the levels of ROS. H_2_O_2_ increases in a wide variety of malignant cells through an imbalance in the antioxidant system (AOS), characterized by high levels of SOD and low levels of H_2_O_2_ detoxifying enzymes, as catalase [4, 13, 14]. Indeed, tumor cell proliferation is inhibited by decreasing H_2_O_2_ through catalase.

Catalase is the main enzyme that dissipates the H_2_O_2_ produced during melanin synthesis [15]. There is a correlation among catalase activity, melanin content and TYR activity [15, 16]. Therefore, considering that H_2_O_2_ inhibits TYR, TYRP1 and TYRP2 [17], the high levels of H_2_O_2_ associated with low catalase activity in melanoma [18–20] would be relevant in the induction of the amelanotic phenotype.

Cell invasion and migration are critical for metastasis and indicate poor prognosis. To migrate, cancer cells develop invadopodia through actin polymerization/depolymerization cycles [21–23]. Metastatic cells present high levels of cofilin-1, a key regulator of actin polymerization. Cofilin-1 severs actin filaments, favoring actin polymerization and so migration [24, 25]. Overexpression of cofilin-1 was associated with loss of cell polarity in colon adenocarcinoma cells [26] and has been used as poor prognosis marker related to metastasis in lung cancer patients [27, 28]. Adenylyl cyclase-associated protein 1 (CAP1) facilitates actin and cofilin-1 recycling, essential for cell migration [29, 30]. CAP1 overexpression was associated with migration in pancreatic cancer cells [31]. However, the behavior of these proteins in melanoma has received little attention.

In this study, a differential compensation to H_2_O_2_ scavenging by catalase overexpression in A375 amelanotic melanoma cells was demonstrated. Cells responded to H_2_O_2_ scavenging by two main strategies: increasing ROS, which resulted in metastasis or maintaining ROS levels, reversing malignant features. Therefore, to overcome the inhibition of cell proliferation induced by catalase overexpression, cells changed the redox status leading to distinct phenotypes ranging from reversion to promotion of melanoma malignancy. Finally, this model allowed the evaluation *in vitro* and *in vivo* of migration related proteins and melanocytic differentiation markers and their association with oxidative stress levels.

## RESULTS

### Characterization of melanoma cells overexpressing catalase

**Figure 1:**
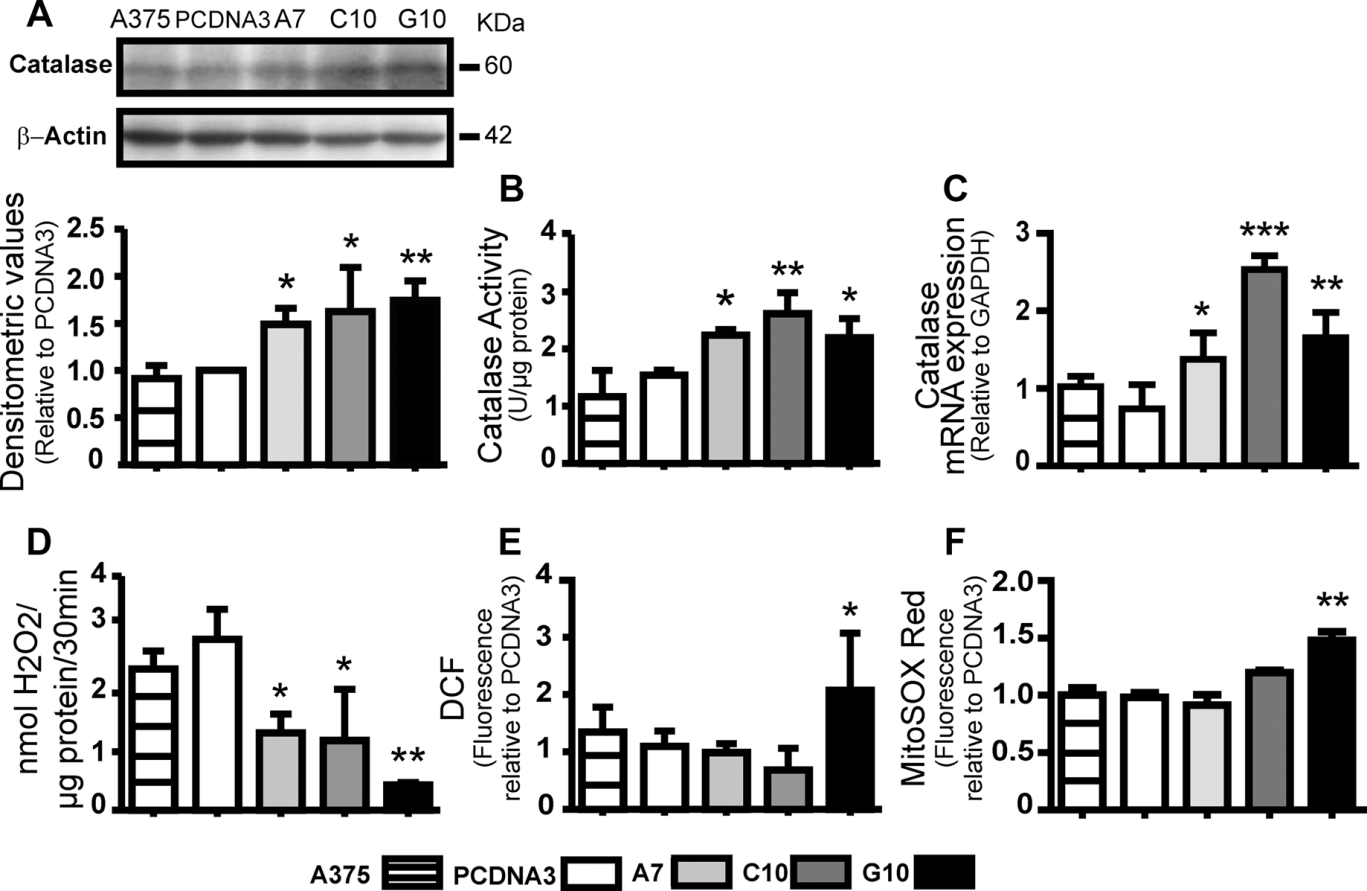
Catalase-transfected A375 cells (A7, C10 and G10) decreased H_2_O_2_ levels but differentially induced ROS levels Control: A375 cells transfected with empty vector (PCDNA3) and not transfected (A375). (**A**) Representative immunoblot images of catalase and β-actin with its densitometric analysis. Actin values were used to normalize samples. Full length blots are included as Supplementary Figure S4. (**B**) Determination of catalase activity by spectrophotometric method. (**C**) Catalase mRNA expression by qPCR (**D**) Quantification of H_2_O_2_ production by Amplex^®^ Red kit. (**E**–**F**) Intracellular ROS levels by quantification of DCF and MitoSOX Red mean fluorescence. Data are expressed as mean ± SD. *p < 0.05; **p < 0.01; ***p < 0.001 vs control (PCDNA3).

Catalase overexpression on human amelanotic melanoma A375 cells gave rise to three clones: A375-A7 (A7), A375-C10 (C10) and A375-G10 (G10). A375 and A375 cells transfected with the empty vector (PCDNA3) were used as control. An increase in catalase activity and expression was observed for all clones vs PCDNA3 (*p* < 0.05) (Figure 1A–1C). H_2_O_2_ levels decreased in all clones when compared to control (*p* < 0.05), with the unexpected increase in G10 ROS production (*p* < 0.05) (Figure 1D–1F). No changes were found neither in glutathione peroxidase activity nor in peroxiredoxin 2 expression (Supplementary Figure S1). These results indicate that catalase overexpression dissipates H_2_O_2_ in all clones and only G10 induced a redox response that increased its basal ROS levels.

### Stable expression of catalase down-regulated cell proliferation parameters

**Figure 2:**
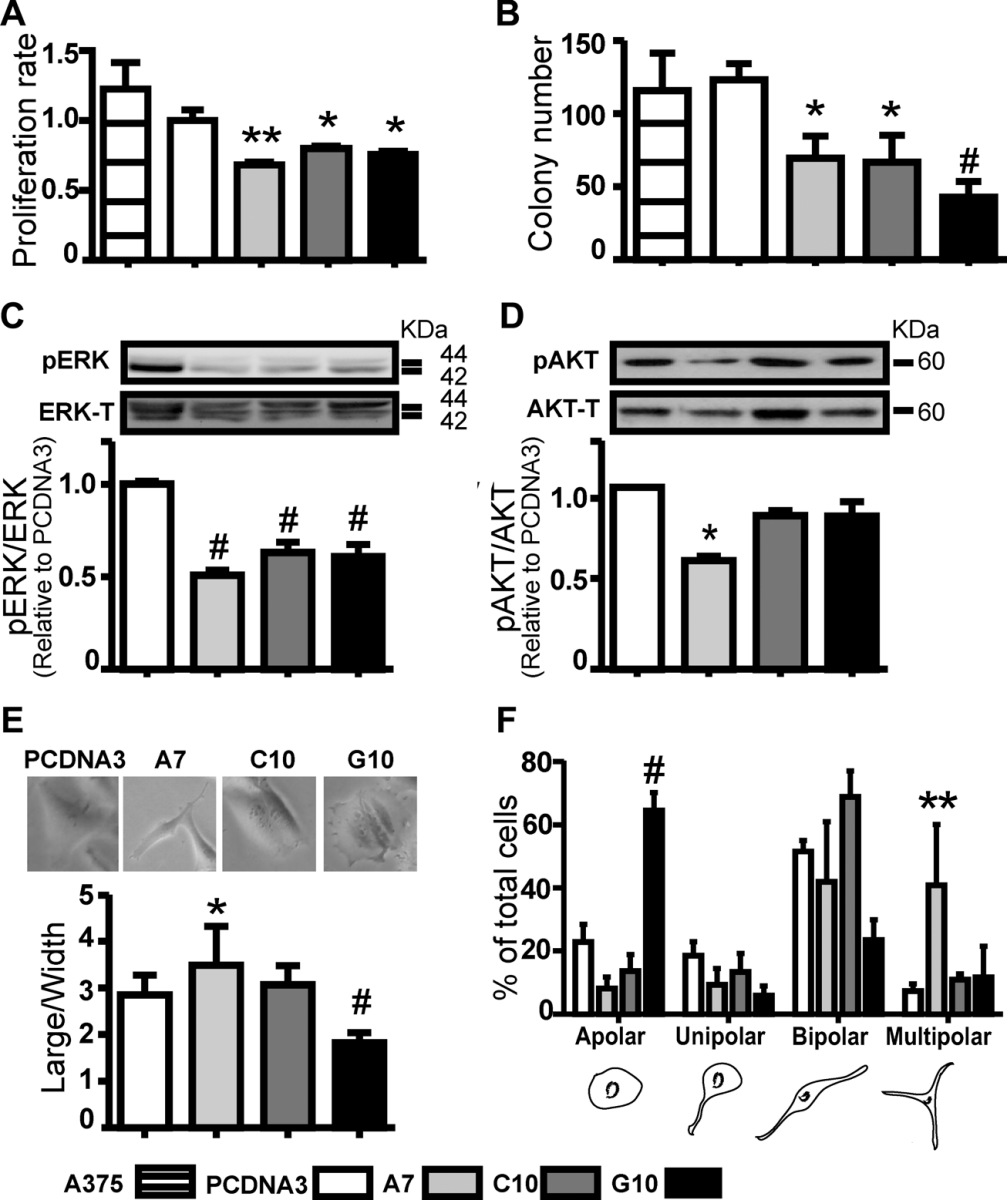
Catalase overexpression down-regulated melanoma cell proliferation parameters and induced different cell polarity degree A375 cells transfected with catalase (A7, C10 and G10) or not transfected (A375) relative to control, A375 cells transfected with empty vector (PCDNA3). (**A**) Cell proliferation rate evaluated by MTT assay. (**B**) Anchorage independent cell growth by soft agar assay. Results represent average number of colony per field, as described in Supplementary Material. (**C**–**D**) Representative immunoblot images of pERK, pAkt, total ERK and Akt. Full length blots are included as Supplementary Figure S4. Densitometric values of pERK or pAkt relative to total ERK or Akt respectively are shown. (**E**) Large to width cell ratio quantification. (**F**) Cell polarity degree was scored as shown in the cartoon below and presented as percentage of total cells scored. Cell scored/condition: 150. Data are expressed as mean ± SD. *p < 0.05; **p < 0.01; ^#^*p* < 0.001 vs control.

Cell proliferation, anchorage-independent cell growth and ERK activity decreased in all clones vs control (*p* < 0.05). AKT activity was significantly lower only for A7 (Figure 2A–2D). Together, these results confirm that catalase overexpression decreases H_2_O_2,_ inhibiting cell proliferation parameters. In view of the similar results between PCDNA3 and A375 cells, most *in vitro* assays were performed using only PCDNA3 as control.

### Different cell polarity degree was induced by catalase overexpression

Cell polarity, particularly melanoma cell dendricity is associated with more differentiated phenotype while its disruption is a hallmark of cancer [32–35]. Increased polarity was found in A7 vs control (*p* < 0.05). On the contrary, a dramatic loss of polarity was observed in G10 vs control (*p* < 0.001). An increase of multipolar cells in A7 and apolar cells in G10 was observed vs control (< 0.01) (Figure 2E–2F). This indicates higher cell differentiation degree in A7, as it resembles melanocytes dendritic like-structure [34–36]. Conversely, the apolar feature of G10 may be associated to amoeboid migration, one of the two main categories of cell movement, characteristic of rounded or ellipsoid cells [25, 37, 38]. To confirm these ideas, melanocyte differentiation features and cell migration parameters were evaluated.

### Catalase overexpression increased melanocyte differentiation

**Figure 3:**
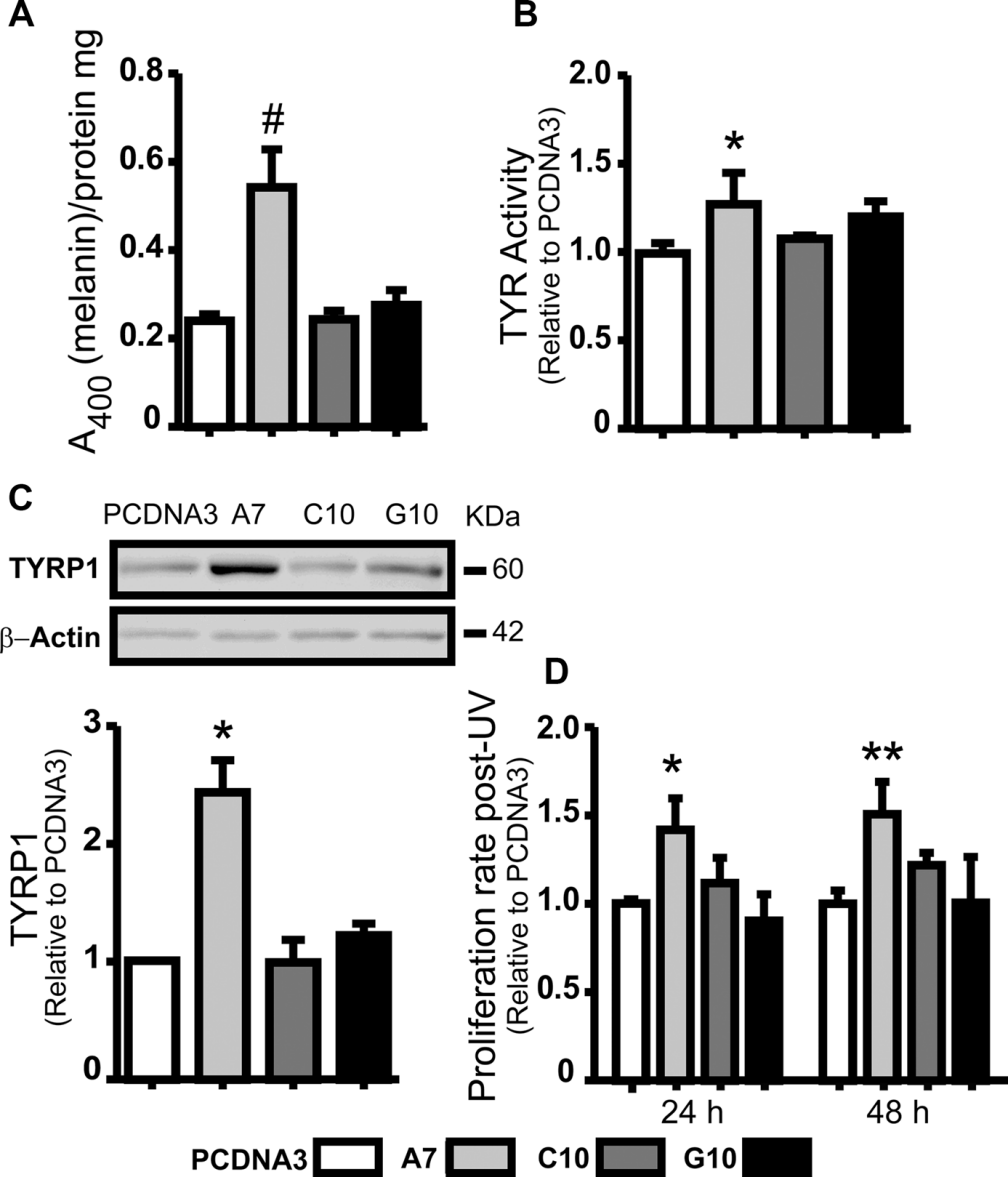
Catalase overexpression induced melanoma cell differentiation A375 cells transfected with catalase (A7, C10 and G10) relative to control, A375 cells transfected with empty vector (PCDNA3). (**A**) Melanin content determination in cell lysates by absorbance at 400 nm. Protein quantification was used to standardize cell samples. (**B**) TYR activity was evaluated indirectly by L-DOPA oxidase activity assay. Dopachrome levels were determined at 492 nm. (**C**) Representative immunoblot images of TYRP1 and β-actin protein expressions with its densitometric analysis. Actin densitometric values were used to normalize samples. Full length blots are included as Supplementary Figure S5. (**D**) Cell proliferation rate 24 and 48 hours post-UV radiation evaluated by MTT assay. Data are expressed as mean ± SD. *p < 0.05; **p < 0.01; ^#^*p* < 0.001 vs control.

Melanogenesis parameters were evaluated to verify whether the multipolarity found in A7 implies its evolution to a normal melanocyte. Results showed an increase in melanin content, TYR activity and TYRP1 expression in A7 compared with the amelanotic control (*p* < 0.05). Moreover, A7 increased the ability to proliferate after UV-A irradiation compared with control (*p* < 0.01) (Figure 3A–3D). Given that melanin protects melanocytes from UV radiation, these results indicate that A7 evolved to a melanotic and differentiated phenotype.

### Catalase overexpression induced melanoma cell migration

**Figure 4:**
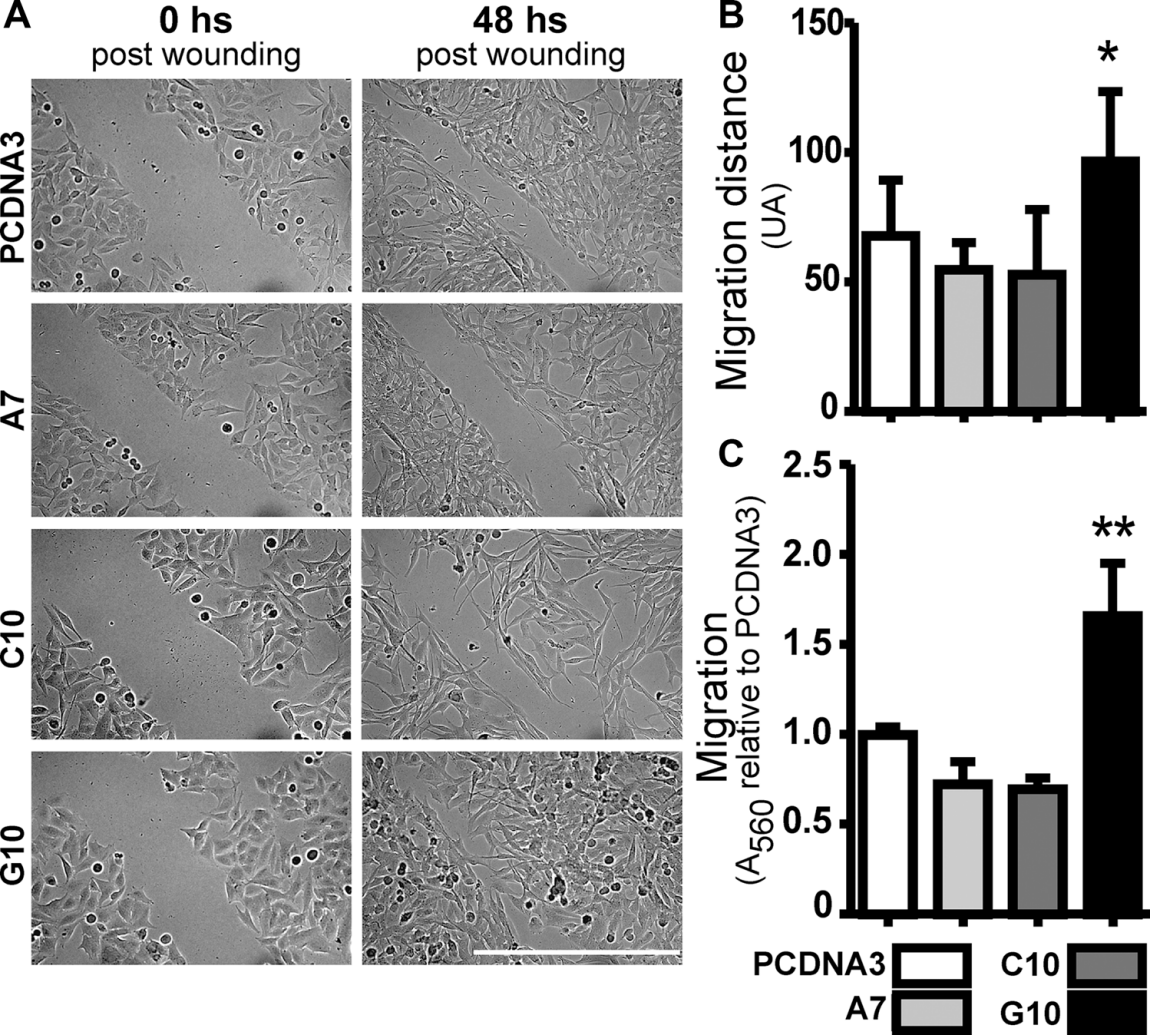
Catalase overexpression induced melanoma cell migration A375 cells transfected with catalase (A7, C10 and G10) relative to control, A375 cells transfected with empty vector (PCDNA3). (**A**) Representative images of wound-healing assay, 0 and 48 hours post-wounding. Bar represents 200 μm. (**B**) Quantification of migration distance calculated as the average difference between the distance in arbitrary units (AU) at time 0 and 48 hours after wounding. (**C**) Evaluation of migration capacity by trans-well assay. Absorbance at 560 nm indicates the amount of cells passing through membrane pores. Data are expressed as mean ± SD. *p < 0.05; **p < 0.01 vs control.

In order to evaluate if G10 had developed the ability to migrate, wound healing (Figure 4A and 4B) and transwell assay (Figure 4C) were performed. Increased migration was observed in G10 vs control and the other clones (*p* < 0.05) (Figure 4A–4C). Note that cells were not synchronized in terms of cell proliferation for wound healing. Therefore, considering that G10 as the other two clones are less proliferative, its increased migration could not be accounted for differences in proliferation rate.

**Figure 5:**
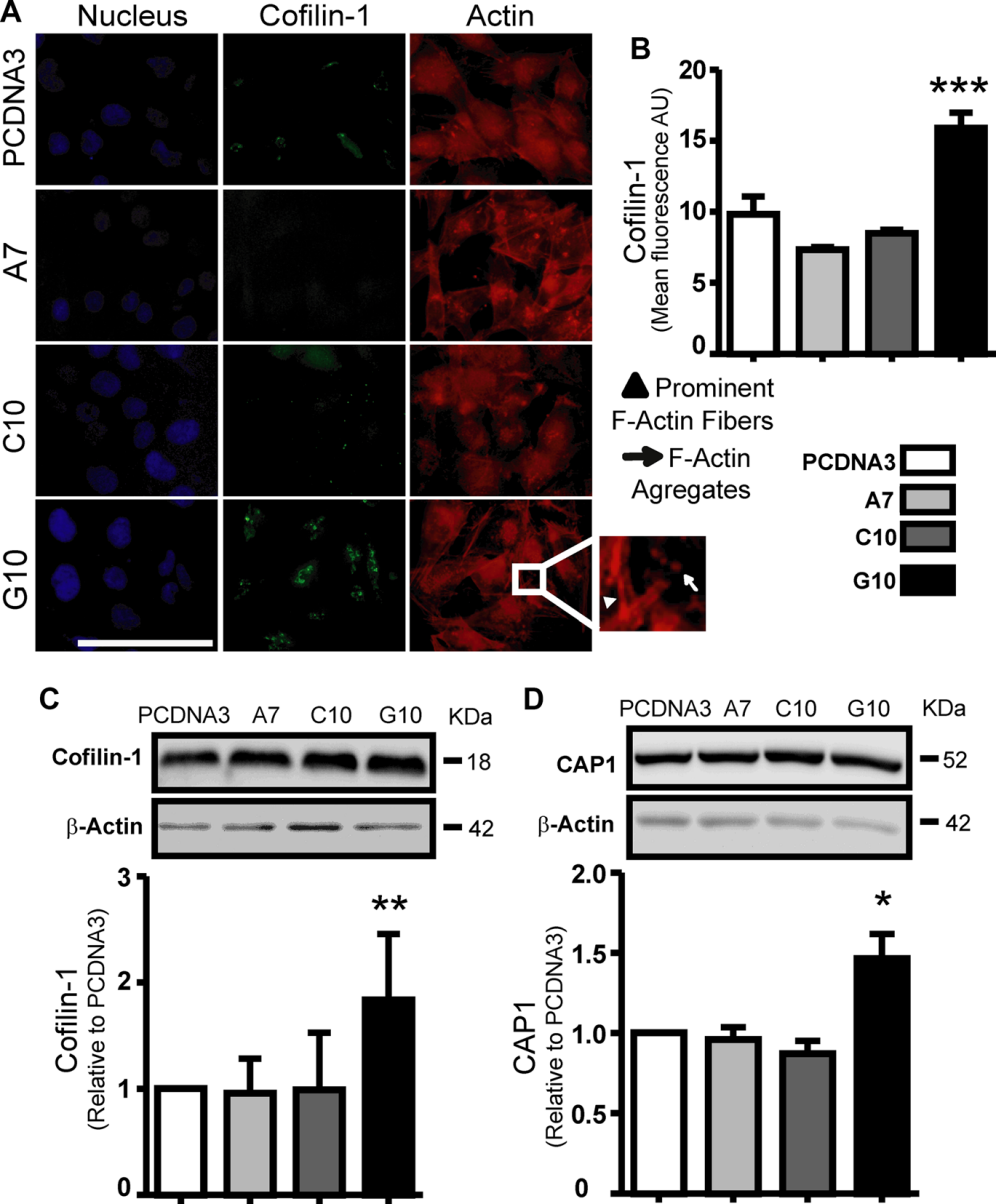
Catalase overexpression induced cofilin-1 and CAP1 expression A375 cells transfected with catalase (A7, C10 and G10) relative to control, A375 cells transfected with empty vector (PCDNA3). (**A**) Representative immunocytofluorescence images of cofilin-1 with their corresponding actin and nuclear staining using rhodamine phalloidin and DAPI respectively. Bar represents 100 μm. Inset image of G10 exhibits its prominent F-actin and F-actin aggregates. (**B**) Quantification of cofilin-1 expression as the mean cell fluorescence arbitrary units (AU). (**C**–**D**) Representative immunoblot images of cofilin-1 and CAP1 protein expression with their respective densitometric analysis. Actin values were used to normalize samples. Full length blots are included as Supplementary Figure S5. Data are expressed as mean ± SD. *p < 0.05; **p < 0.01; ***p < 0.001 vs control.

Actin polymerization state, cofilin-1 and CAP1 were evaluated because of their relevance in cell migration. Increased actin fibers (F-actin) and F-actin aggregates in the leading edge of G10 cells were observed (Figure 5A). Furthermore, cofilin-1 and CAP1 expression increased in G10 vs control (*p* < 0.05) (Figure 5A–5D). CAP1 was differentially located in protrusions of G10 cells with fiber actin (Supplementary Figure S2). These results indicate an increased actin polymerization state in G10 that favors its ability to migrate.

### Differential tumorigenicity was induced by catalase overexpression

**Figure 6:**
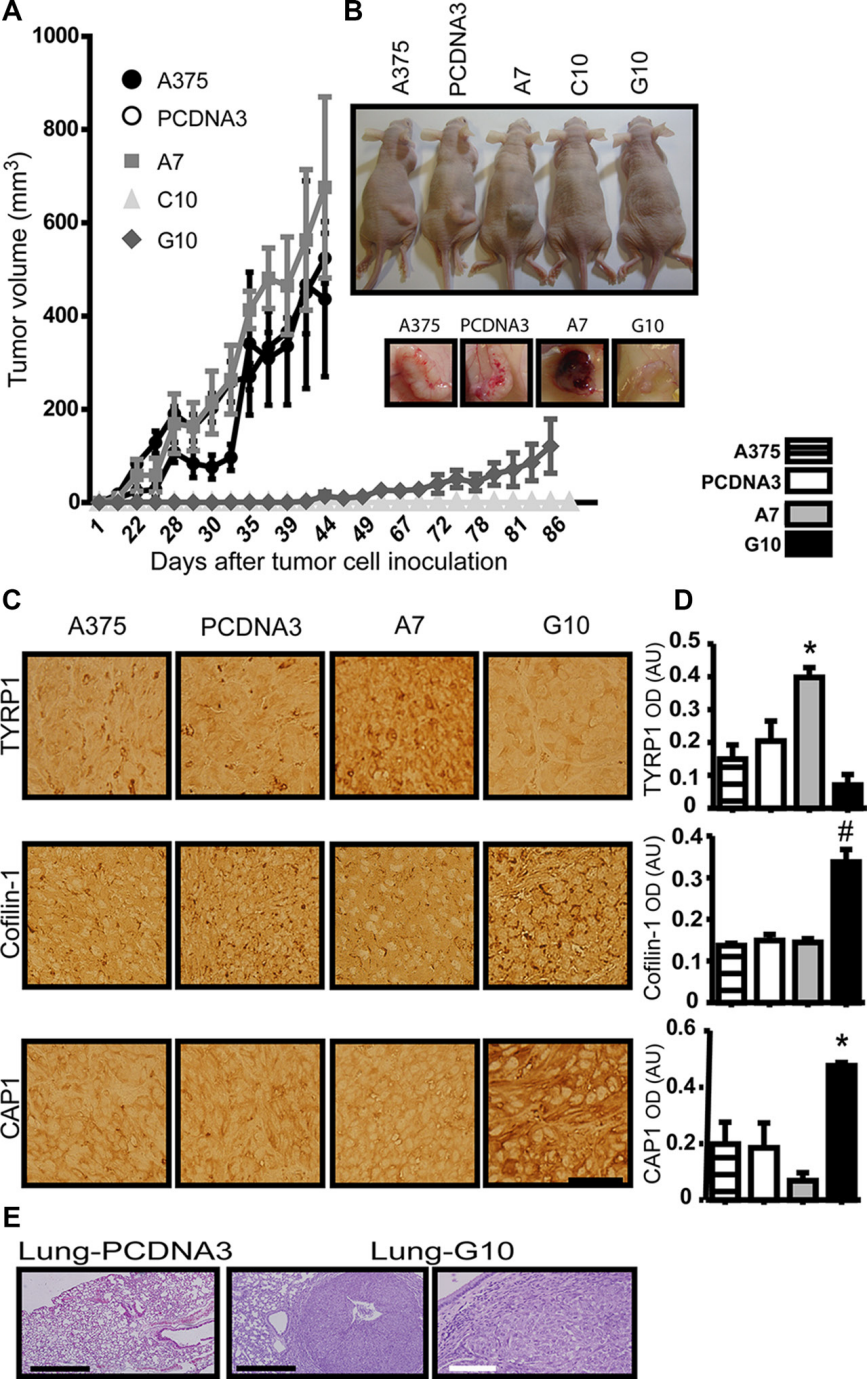
*In vivo* validation of *in vitro* results in tumors induced by melanoma cells overexpressing catalase Nude mice inoculated with A375 cells transfected with catalase (A7, C10 and G10), with empty vector (PCDNA3) or not transfected (A375). (**A**) Tumor growth curves over time post-inoculation by monitoring tumor volume throughout time. (**B**) Representative images of mice inoculated with each cell type and the respective tumors of the tumorigenic ones: A375, PCDNA3, A7 and G10. (**C**) Representative images of TYRP1, cofilin-1 and CAP1 IHC detection in tissue sections of tumors induced by A375, PCDNA3, A7 and G10 cells. Bar represents 50 μm. (**D**) Quantification of IHC by optic densitometric (OD) analysis of images. Data are expressed as mean ± SD. *p < 0.05; ^#^*p* < 0.001 vs control (PCDNA3). (**E**) Lung tissue sections stained with H&E. Representative images of G10 lung nodules indicate its metastatic ability. Control lung tissue section is also shown. Black bars represent 500 μm and white bar 100 μm.

Regarding tumorigenesis, A7 induced pigmented tumors denoting increased differentiation, although they grew similar to controls (A375 and PCDNA3). On the contrary, tumors from G10 had more than 40 days delay in growth with significant decrease in size when compared to controls, even allowing to grow the same time since they appear, consistent with it less *in vitro* proliferation rate. C10 never induced tumors, even after 6 month since inoculation. No differences were found between controls (Figure 6A–6B).

Representative images of H&E stained tumor tissue sections are shown (Supplementary Figure S3). High cell proliferation can be observed in tumors induced by controls and A7, but A7 tumors present areas with brownish granules of melanin deposits (Supplementary Figure S3), supporting the *in vitro* results of melanin synthesis and differentiation. On the other hand, G10 tumors were mostly necrotic. Note that as C10 did not induce tumors, no H&E/immunohistochemistry was performed.

Besides, an increased expression of TYRP1 in A7 (*p* < 0.05) and of cofilin-1 and CAP1 in G10 compared with controls were found (*p* < 0.05) (Figure 6C–6D). These results confirmed *in vivo* that stable overexpression of catalase induced either melanogenesis or migration, which could be associated with the unchanged levels of ROS in A7 or its increased levels in G10.

Considering the migration ability of G10, *in vivo* assay of tumor metastasis was performed. Pulmonary metastatic nodules were induced only by G10, indicating its evolution to a more aggressive phenotype (Figure 6E) and reinforcing the idea that its increased ROS levels could be associated with its malignant phenotype.

## DISCUSSION

This work shows that overexpression of catalase in A375 melanoma cells inhibits proliferation in agreement with previous results [4, 39]. Besides, anchorage-independent cell growth inhibition was also found. However, the response to catalase overexpression led to quite distinct phenotypes that ranged from reversion to promotion of malignancy. When cells responded to H_2_O_2_ decrease increasing ROS levels, migration and metastasis were induced (G10), but when ROS levels did not change, less aggressive (A7) or nonmalignant (C10) phenotypes were generated.

Tumors progress with continuous ROS increase due to an AOS imbalance, which is characterized by low levels of catalase and glutathione peroxidase [4, 13, 39, 40]. Given that glutathione peroxidase activity and peroxiredoxin 2 expression did not change in these clones, H_2_O_2_ levels mainly decreased by the overexpression of catalase.

Besides, ERK1/2 activation increases proliferation [41]. Hydrogen peroxide can increase ERK1/2 activity by the oxidation of its phosphatases [42, 43]. These studies could explain how the decrease of H_2_O_2_ in these clones reduced its ERK1/2 activity together with cell proliferation and anchorage-independent cell growth.

Dedifferentiation/differentiation are associated with more or less aggressive tumors [32, 33]. Clone A7 enhanced polarity with melanoma cell dendricity, a parameter of melanoma differentiation [34, 35]. Moreover, the activation of melanogenic pathway was observed in A7 by the increase in melanin content, tyrosinase activity and TYRP1 expression. The increase of catalase activity correlates with melanin content [16]. Pigmentation and melanocyte differentiation markers (tyrosinase and TYRP1) decreased by high H_2_O_2_ levels [15, 17]. Furthermore, tyrosinase and TYRP1 correlates inversely with tumor stage and its progression to the amelanotic phenotype [44–46]. Supporting the increased UV-A tolerance of A7, melanocytes offset deleterious effects of UV radiation by melanin and catalase [15, 47]. Therefore, these previous reports support how A7 turned to a differentiated and therefore less aggressive phenotype.

Only A7 showed a significant decrease of Akt activity. In this regard, inhibition of Akt in melanoma cells increases melanogenesis pathway, by inducing MITF, meanwhile the inhibition of ERK did not [48, 49]. These previous studies could explain why melanogenesis was induced in A7 but not in C10 and G10. Thus, the induction of differentiation in A7 by catalase overexpression could be mediated by the inhibition of Akt activation, reinforcing its participation in the melanogenesis pathway.

Clone G10 showed a clear change towards dedifferentiation, revealed by the loss of polarity, characteristic of migration and metastasis progression [25, 38]. The fact that G10 increased ROS levels, although decreased H_2_O_2_, suggest that other ROS are responsible for the oxidative status of G10. The expression of proteins related to migration and metastasis induced by ROS [10] can explain the ability of G10 to migrate and metastasize. Therefore, switching to a migrating phenotype could be “an escape program” from high ROS levels [10]. In a mouse melanoma model, the antioxidant N-acetylcysteine (NAC) increased lymph node metastases but had no impact on the number and size of primary tumors [50]. These results agree with the metastatic ability and the small primary tumor size of G10.

Melanoma cells invade and metastasize by amoeboid or mesenchymal migration [37, 38, 51]. Amoeboid migration, characteristic of round cells, is induced by actin-rich fillopodia at the leading edge of cells facilitating protrusions [24, 37], as observed in G10. Increase of cofilin-1 and CAP1 was found in G10. These proteins favors actin polymerization and so migration [29]. Cofilin-1 is a key factor in tumor cell migration and metastasis, being overexpressed in a wide variety of cancers [24, 26, 27, 52]. Cofilin-1 is activated by dephosphorylation through the phosphatase Slingshot-1L (SSH-1L) [53, 54]. High levels of ROS activate cofilin-1 through the release of SSH-1L from its regulatory protein when oxidized by ROS, increasing cell migration [55]. Thus, high ROS production found in G10 could activate cofilin-1 by dephosphorylation. Colocalization of CAP1 with F-actin in the leading edge of cells has been involved in metastasis. Moreover, repression of cofilin-1 or CAP1 in metastatic cells inhibited migration [25, 31, 56, 57]. Therefore, not only the high levels of CAP1 and cofilin-1, but also the high ROS production found in G10 could be key events that promote migration and metastasis.

*In vivo* assays validated *in vitro* results. Tumorigenicity was completely inhibited in C10 and decreased in G10, consistent with the inhibition of proliferation and the antitumor effect of catalase [14, 58]. Even though A7 was tumorigenic, tumors were pigmented with increased expression of TYRP1. This confirms that A7 is a differentiated melanoma, which could be associated with a less aggressive phenotype. On the contrary, the increase of cofilin-1 and CAP1 together with the metastasis induced by G10 *in vivo* supports the fact that G10 evolved to an aggressive phenotype.

In conclusion, these cells faced H_2_O_2_ dissipation by two main strategies: maintaining or increasing ROS levels. Malignant features were reversed by catalase overexpression when ROS levels remained unchanged as demonstrated in C10 and A7. Moreover, when H_2_O_2_ scavenging was associated to Akt inactivation, melanogenesis pathway was triggered, as shown in A7 where TYRP1, tyrosinase and melanin content increased. On the other hand, when the response to catalase overexpression led to high ROS levels, amoeboid migration and actin polymerization were induced by both increasing cofilin-1 and CAP1 proteins, which are the driving force of migration and metastasis, as demonstrated in G10.

The oxidative stress conditions that exist within the tumor and its microenvironment exert strong adaptive pressure on cancer cells, which in order to survive, promote the expression of ROS pathways reprogramming the transcriptome, proteome and metabolism [59]. Considering that H_2_O_2_ scavenging inhibits cell proliferation, we propose herein that melanoma cells triggered different responses to reach a redox homeostasis overcoming the effects on survival induced by catalase. Consistent with these idea, gene expression microarrays analysis of A7 and G10 [60] confirmed both *in vitro* and *in vivo* results and revealed coexpressed AOS genes upregulated in A7 and downregulated in G10, which could explain the different redox responses generated by each clone. Thus, even though it must be necessary to ascertain the results presented here in other cellular models, it is noteworthy that overexpression of an antioxidant enzyme, such as catalase, could favor not only the reversion of malignant processes but also the progression to a worse outcome. Therefore, taking into account that antioxidants can both, prevent or promote cancer progression [50, 61–63], it is necessary to reconsider the use of antioxidants as a strategy against cancer without studying compensatory responses of the AOS.

In addition, the development of these stable clones with distinct characteristics in terms of antioxidant metabolism, pigmentation, migration and metastasis provides a new melanoma model for studying mechanisms associated with differentiation/dedifferentiation and malignant progression under the same genetic background. Finally, this work allowed proposing TYRP1, cofilin-1 and CAP1 as potential markers to detect melanomas with varying degrees of aggressiveness.

## MATERIALS AND METHODS

### Cell culture and catalase overexpression melanoma model

Low-passages human amelanotic melanoma cell line A375 was kindly donated by Dr. E. Medrano (Huffington Center on Aging, Departments of Molecular & Cellular Biology and Dermatology, Baylor College of Medicine, Houston, Texas, USA). All the experiments with these cells were performed with less than 5 passages from thawing. Cells were cultured as previously described [4]. Stable transfected cells were maintained in identical conditions with 700 μg/ml geneticin (Sigma). Cells were regularly tested to be mycoplasma-free.

A375 cells were transfected with pcDNA3 vector harboring the human catalase cDNA (p-CAT) using Lipofectamine 2000 (Invitrogen) [4]. Stable transfectants were selected in medium with 1500 μg/ml geneticin. Three resistant clones A375-A7, A375-C10 and A375-G10, referred in this work as A7, C10 and G10 respectively, were obtained by clonal dilution. Given that catalase inhibits cell proliferation only three clones were possible to obtain. Transfected cells with empty vector were used as negative control and named A375-PCDNA3, referred here as PCDNA3.

### *In vitro* experiments

### Determination of catalase and redox parameters

Catalase mRNA expression was determined by quantitative real-time PCR (qPCR). Forward and reverse primer sequences were: ʹcctttctgttgaagatgcggcgʹ3 and 5ʹggcggtgagtgtcaggatagʹ3 respectively. Cycling conditions are detailed in Supplementary Table S1. For protein expression western blot was performed using anti-catalase antibody (Calbiochem, EMD Milipore) and the detection of β-actin (Sigma antibody) as loading control. Catalase activity was measured using a spectrophotometric assay [4].

The intracellular levels of H_2_O_2_ were evaluated by spectrophotometry according to Amplex^®^ Red kit (Invitrogen). A standard curve of H_2_O_2_ was performed. Results were expressed as H_2_O_2_ nmol released in 30 min per protein μg.

The levels of ROS were determined by 2ʹ,7ʹ-dichlorodihydro-fluorescein diacetate (DCFH-DA, Sigma) assay [64] and MitoSOX^™^ Red (Invitrogen) [65].

Glutathione peroxidase (GPx) activity was measured by an indirect spectrophotometric assay [66, 67]. Peroxiredoxin 2 expression was detected by western blot using anti-peroxiredoxin 2 (Sigma) and β-actin antibody (Sigma) as loading control.

### Determination of cell proliferation parameters

Proliferation was determined by MTT assay [68]. Anchorage independent cell growth was evaluated by soft agar colony formation assay.

The levels of the active form of ERK1/2 and Akt (p-ERK1/2 and p-Akt) related to the total form of these proteins were detected by western blot as a measure of their activity.

### Cell polarity

Cell polarity was evaluated by measuring the cell length/width ratio (L/W) and cells were classified as apolar, unipolar, bipolar or multipolar as shown in Figure 2F [25].

### Determination of melanogenesis parameters

Melanin content was evaluated in cell lysates by spectrophotometry at 400 nm and expressed per protein mg. Tyrosinase activity was assayed by measuring the L-3, 4-dihydroxyphenylalanine (L-DOPA) oxidase activity. The dopachrome levels were determined at 492 nm [69]. TYRP1 expression was assessed by western blot using anti-TYRP1 antibody (Abcam) and β-actin antibody (Sigma) as loading control.

### Cell response to UV-A radiation

After 24 hours culture, 5000 cells were irradiated with 29 kJ/m^2^ total irradiation dose [70].

Cell proliferation was evaluated 24 and 48 hours post irradiation through MTT assay.

### Cell migration: Wound healing and transwell assay

A scratch in the cell monolayer was performed and migration extent was evaluated at 48 hours after healing. Transwell migration assay was performed using the CytoSelect^™^ Cell Migration Assay Kit, according to manufacturer's indications (Cell Biolabs, Inc).

### Evaluation of cofilin-1, CAP1 and actin polymerization state

Standard immunocytofluorescence and western blot using anti-cofilin-1 or anti-CAP1 antibodies (Abcam) were performed. Detection of β-actin (Sigma antibody) was used as loading control in western blot. Rhodamine-phalloidin (Sigma) staining was used for actin visualization.

### *In vivo* experiments

Six-week-old female athymic nude (nu/nu) mice were obtained from the CNEA animal facility (Argentina). Animals were kept under conventional housing conditions. To evaluate the *in vivo* effect by catalase overexpression, tumors and metastasis were induced by injecting 2.5 × 10^6^ cells subcutaneously in one flank or through the tail vein respectively, using 5 animals per experimental condition. As control of cell viability of tail vein inoculated cells at the moment of injection, cells were inoculated subcutaneously in 2 nude mice per condition. All procedures were carried out according to the Laboratory Animal Care and Use guidelines from CNEA and to the relevant international guidelines.

### Evaluation of tumor growth and metastasis

Tumor growth was evaluated by determining tumor volume every other day during up to 3 month. To study the development of metastasis, mice autopsy was conducted. Tumors and organs with naked eyes metastasis were processed for histopathological examination (H&E) and immunohistochemistry.

### Immunohistochemistry detection of TYRP1, cofilin-1 and CAP1

Standard immunohistochemistry was performed using proper antibodies and Super Sensitive IHC Detection Systems (BioGenex) or Picture-MAX Polymer (Invitrogen) kits following manufacturer's instructions. Rabbit anti-TYRP1, cofilin-1 and CAP1 antibodies (Abcam) were used.

### Statistical analysis

Significant differences between group means were assessed by one-way analysis of variance followed by Dunn's multiple comparisons test. For tumor growth analysis, when parametric tests were not possible to apply, Friedman test for matched observations was performed followed by Dunnett's multiple comparisons test. *P*-values lower than 0.05 were considered significant for all tests.

Detailed description of all methods is included in Supplementary Materials.

## SUPPLEMENTARY FIGURES AND TABLES


