# Supplementary material for: Reprogramming human A375 amelanotic melanoma cells by catalase overexpression: Reversion or promotion of malignancy by inducing melanogenesis or metastasis
Source: Oncotarget. 2016 May 7;7(27):41142–53. doi: 10.18632/oncotarget.9220 (PMC5173048; doi:10.18632/oncotarget.9220)
Supplement: Supplementary file 1 [file oncotarget-07-41142-s001.pdf]

Reprogramming human A375 amelanotic melanoma cells by catalase overexpression: Reversion or promotion of malignancy by inducing melanogenesis or metastasis

Supplementary Materials

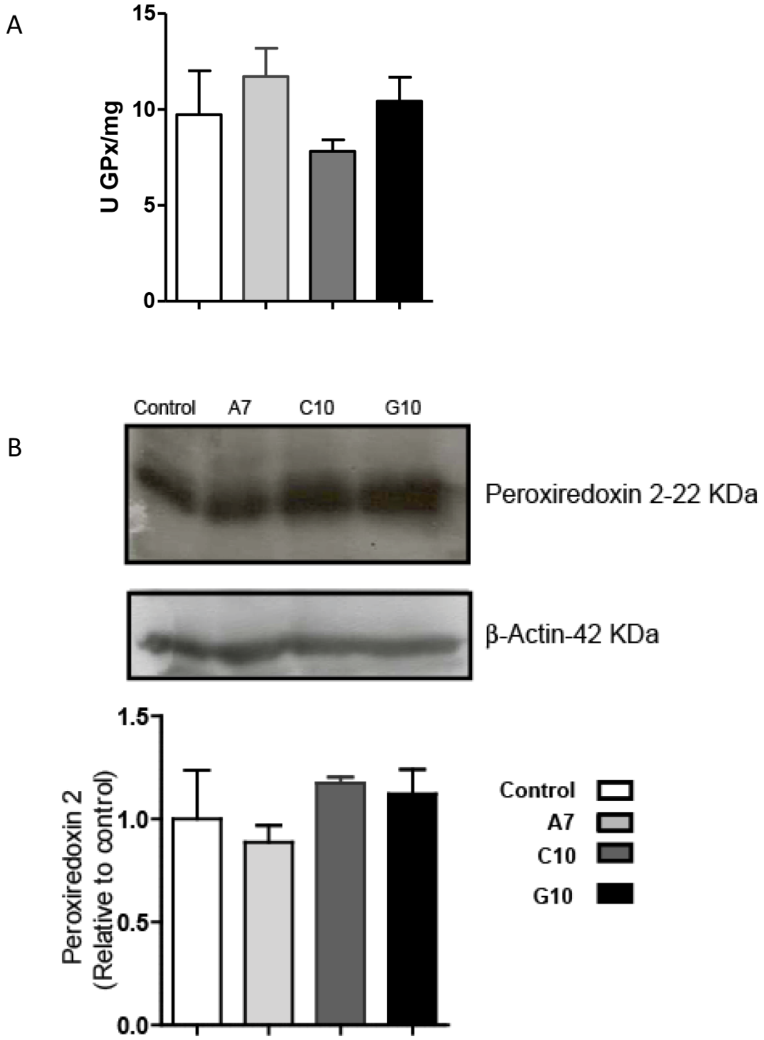

Supplementary Figure S1: Glutathione peroxidase (GPx) activity and Peroxiredoxin 2 expression did not change in catalase-transfected A375 cells (A7, C10 and G10). Control: A375 cells transfected with empty vector (PCDNA3). **(A)** Determination of GPx activity by indirect spectrophotometric method. **(B)** Representative immunoblot images of peroxiredoxin 2 and β-actin with its densitometric analysis. Actin values were used to normalize samples. Data are expressed as mean ± SD. No significant differences were found between samples.

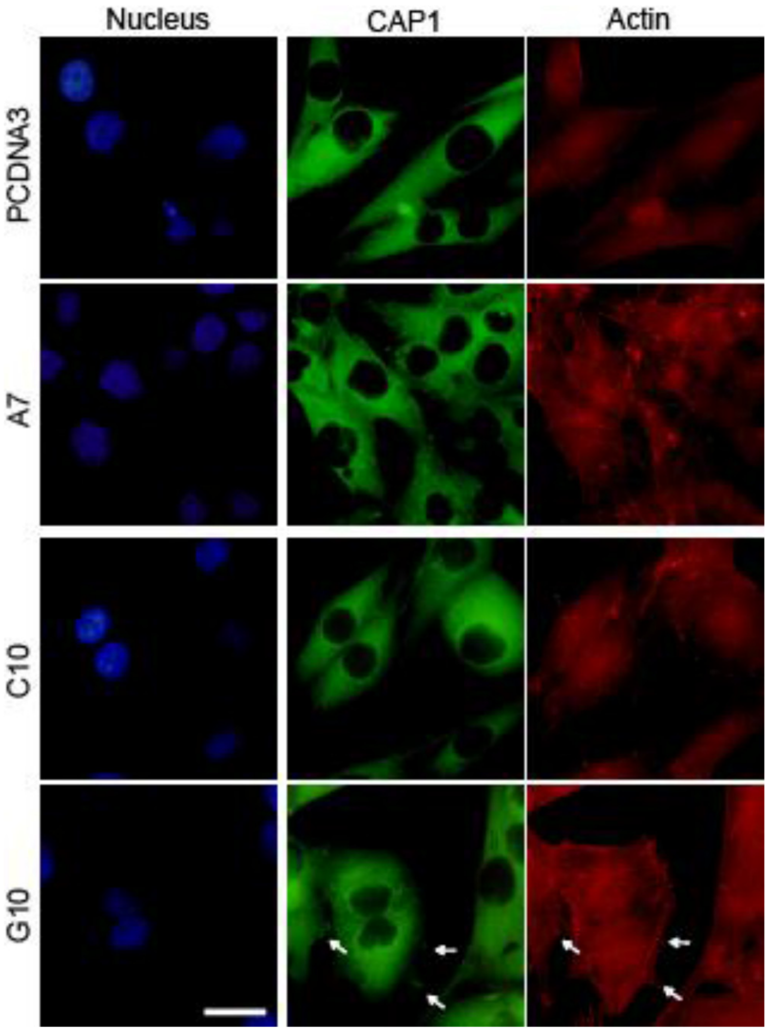

Supplementary Figure S2: Catalase overexpression induced differential localization of CAP1. A375 cells transfected with catalase (A7, C10 and G10) and control, A375 cells transfected with empty vector (PCDNA3). Representative immunocytofluorescence images of CAP1 with their corresponding actin and nuclear staining using rhodamine phalloidin and DAPI respectively. Bar represents 25 µm. Arrows indicates higher localization of CAP1 and actin aggregates in G10 protrusions.

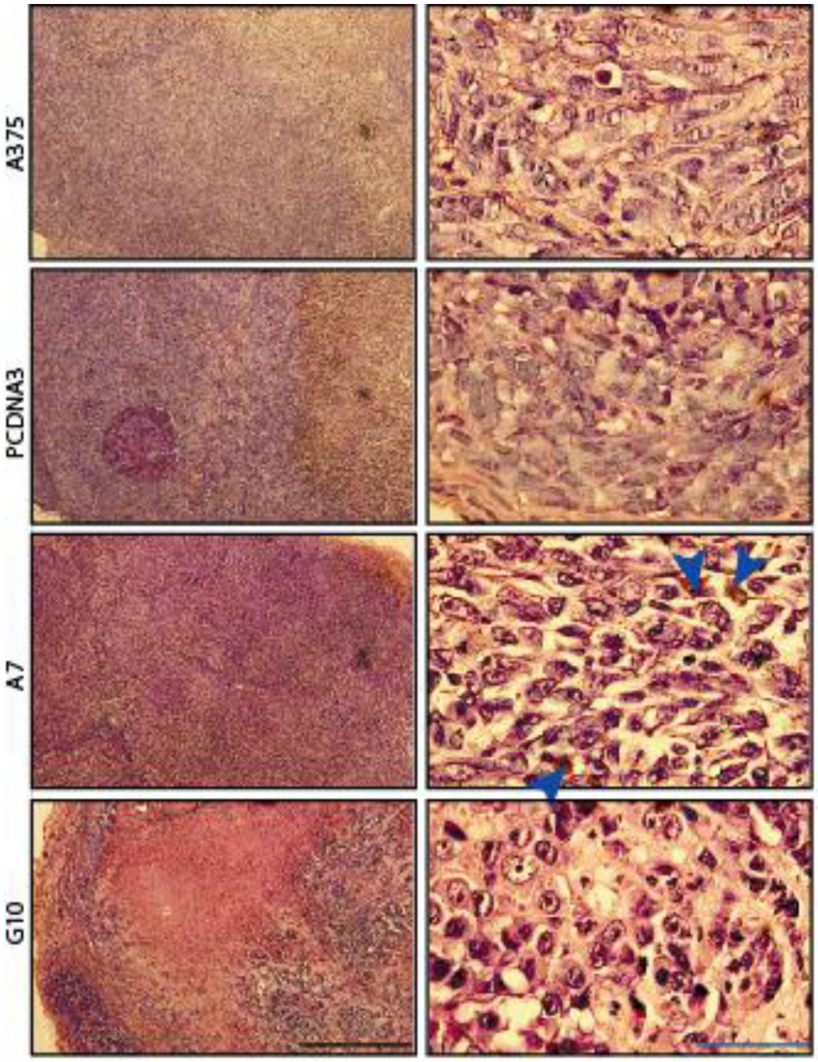

Supplementary Figure S3: *In vivo* validation of *in vitro* results in tumors induced by melanoma cells overexpressing catalase. Representative IHC images of tissue sections stained with H&E of tumors induced by A375, PCDNA3, A7 and G10 cells. Arrowheads in A7 indicate melanin deposition. Black bar represent 500 µm and blue bar 100 µm.

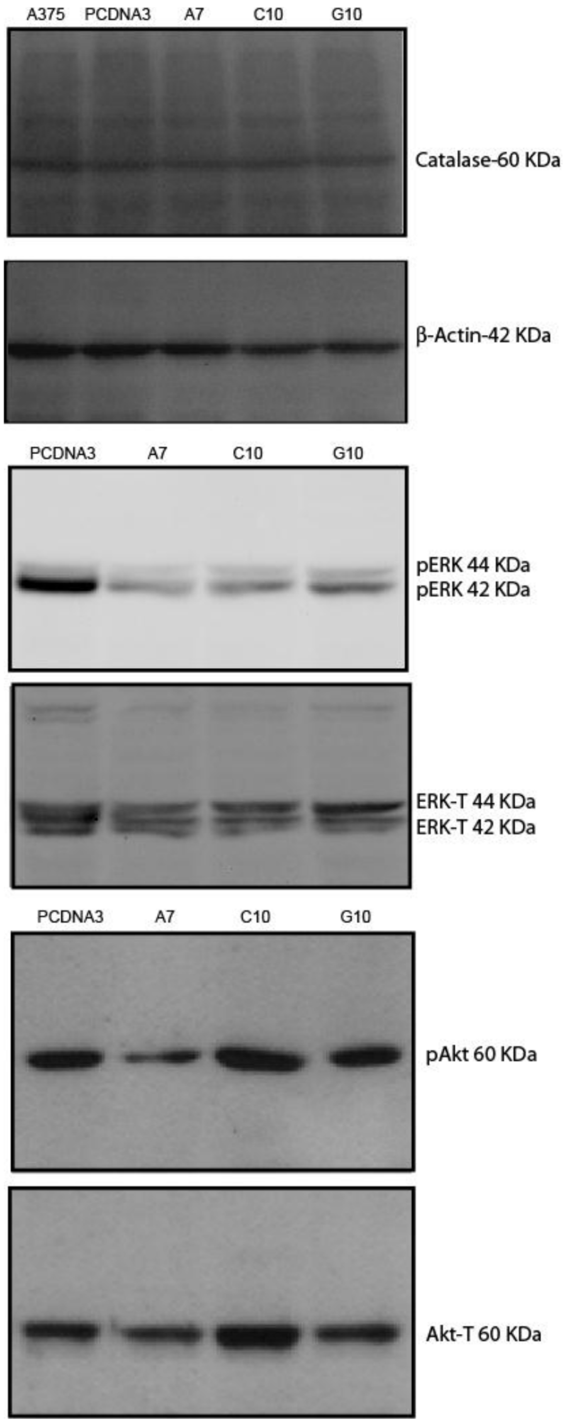

Supplementary Figure S4: Representative full length immunoblot images of catalase with their corresponding actin in A375, PCDNA3, A7, C10 and G10 cells and pERK/ERK-T and pAKT/AKT-T in PCDNA3, A7, C10 and G10 cells.

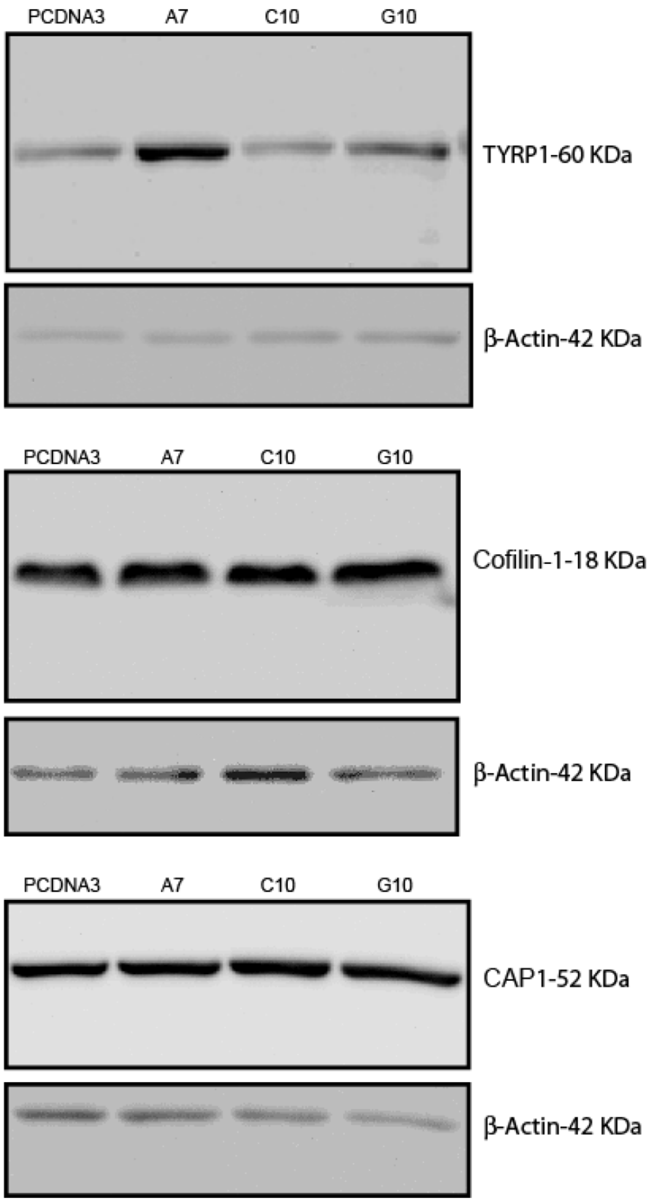

Supplementary Figure S5: Representative full length immunoblot images of TYRP1, Cofilin-1 and CAP1 with their corresponding actin in PCDNA3, A7, C10 and G10 cells.

MATERIALS AND METHODS

Catalase expression and activity

The catalase mRNA expression was determined by quantitative real-time PCR (qPCR). Total RNA samples were isolated from cultured cells using RNAspin Mini RNA Isolation Kit (GE Healthcare), following manufacturer instructions. RNA quantity and quality were determined by NanoDrop2000 photometer (Thermo). RNA integrity was assessed evaluating the ~2:1 ratio of 28S:18S bands in 1% agarose gel electrophoresis. Four biological replicates were used. Syntheses of cDNA from the extracted RNA samples were performed using polymerase reverse transcriptase (SuperScript™ II, Invitrogen) following manufacturer indications. For qPCR 15 µl final reaction volume, 7 µl Master mix (Maxima SYBR Green qPCR Master Mix, Biotium 2X), 0.2 µl primers 10 µM, 3 µl of cDNA 1:50 and free nuclease water (Biodynamics) were used. Sequences of primers (Invitrogen) and cycling conditions are detailed in Supplementary Table S1. Reactions were carried out in a QIAGEN's qPCR cycler. The Pfaffl mathematical model for relative quantification was used to calculate the mRNA expression level (normalized to GAPDH) [8].

Catalase protein expression was determined by western blot. Protein extracts were prepared by lysing cells with RIPA buffer (Sigma) in presence of Halt protease and phosphatase inhibitor cocktail (Thermo Scientific). Samples were separated by 10% SDS polyacrylamide (Promega) gel electrophoresis, transferred to nitrocellulose membranes (Hybond ECL Membrane, Amersham, GE Healthcare) and immunoblotted by rabbit anti-catalase antibody 1:2000 (Calbiochem, EMD Millipore). For loading control, rabbit β-actin detection (Sigma antibody, 1:1000) was performed. The primary antibodies were detected using horseradish peroxidase-linked donkey anti-rabbit IgG 1:2000 (Amersham, GE Healthcare) and visualized by chemiluminescence detection solution (Buffer Tris 0.1 M pH 8.6, luminol 1.24 mM, p-coumaric 0.196 mM, H_2O_2 3%) [1]. Quantification was performed by densitometric scanning with the ImageJ software. Three independent experiments were performed in duplicate each time.

The catalase activity was measured using a spectrophotometric assay as described by Aebi et al. [2]. Briefly, cells grown in 100-mm dishes at 70% confluence were scraped, homogenized in sodium phosphate buffer 50 mM, pH 7, lysed by sonication and clarified after centrifugation at 15000 g. To start the reaction 1 ml of phosphate buffer 50 mM, pH 7 containing H_2O_2 30 mM was mixed with 200 µg proteins. The decomposition of H_2O_2 was followed directly by the decrease in absorbance at 240 nm. A unit of catalase is defined as the disappearance of 1 µmol H_2O_2/min at 25 ºC (E = 39.4 M^{-1} cm^{-1}). Enzymatic activity was expressed in units/mg protein. Protein concentration was determined by Lowry. Three independent experiments were performed with triplicates per condition.

Glutathione peroxidase activity

GPx activity was determined by an indirect spectrophotometric assay [3, 4]. Briefly, cells were grown in 100-mm dishes at 70% confluence, scraped in PBS, lysed by three cycles of frozen-thawed and clarified after centrifugation at 15,000 g. Protein concentration was determined by Lowry. Proteins samples (40 µg/40 µl), 20 µl NADPH 5 mM in NaHCO3 0.1% and 160 µl phosphate buffer 20 mM, pH 7.7 were placed in a 96-well plate and incubated 10 min at 37ºC. Then, 10 µl reduced glutathione (GSH) 40 mM and 10 µl glutathione reductase (GR) 40 U/ml freshly prepared were added. Absorbance at 340 nm was measured for 3 min at 37ºC. To start the reaction 20 µl tert-butyl hydroperoxide 70% in water, protected from light, was added and absorbance again measured during 6 min. A unit of glutathione peroxidase is defined as the amount of enzyme able to produce 1 µmol $NADP^+$/min at 37ºC (E = 6.22 mM^{-1} cm^{-1}) [4]. Enzymatic activity was expressed in units/mg protein. Three independent experiments were performed with triplicates per condition.

Peroxiredoxin 2 expression

The levels of peroxiredoxin 2 expression was determined by western blot as described above, using rabbit anti-peroxiredoxin 2 antibody 1:2000 (Sigma) and rabbit β-actin antibody 1:1000 (Sigma) as loading control. Both antibodies were detected by a horseradish peroxidase-linked sheep anti-rabbit IgG 1:1000 (Amersham, GE Healthcare). Two independent experiments were done in duplicate each time.

Determination of H_2O_2 and ROS

The intracellular levels of H_2O_2 were evaluated by spectrophotometric assay according to Amplex® Red kit (Invitrogen). Briefly, a total of 20,000 cells were seeded in 24-well plate. After 48 hours of culture, cells were washed with PBS and sodium phosphate buffer 50 mM pH 7.4 was added. The reaction started with Amplex® Red solution prepared according to manufacturer instructions (Amplex® Red reagent, 10-acetyl-3, 7-dihydroxyphenoxazine in DMSO and 0.2 U/ml horseradish peroxidase, HRP). Cells were incubated at 37°C, 5% CO_2, during 30 min and absorbance at 560 nm was measured in a plate reader. To quantify, a standard curve of H_2O_2 was performed in parallel. A second 24-well plate was seeded under the same conditions and cells were lysed with NaOH 0.1 N plus freeze-thaw cycles for protein quantification. Results were expressed as H_2O_2 nmol released in 30 min

Supplementary Table S1: Primers sequence for qPCR

Gene	5′	Forward Primer / Reverse Primer	3′	40 Cycles Tm (°C/s)	40 Cycles Extension (°C/s)
CAT		cctttctgttgaagatgcggcg		60/30	72/20
		ggcggtgagtgtcaggatag			
GAPDH		cccactcctccacctttgac		60/20	72/20
		cataccaggaaatgagcttgacaa			

per protein μg. Protein concentration was determined by Lowry. Three experiments were performed with triplicates per each condition.

The levels of ROS were determined by 2′,7′-dichlorodihydro-fluorescein diacetate (DCFH-DA, Sigma) [5] and 3, 8-phenanthridinediamine, 5-(6′-triphenyl-phosphoniumhexyl)-5, 6-dihydro-6-phenyl, (MitoSOX™ Red, invitrogen) [6] assays. For DCFH-DA, cells grown at 70% confluence were washed with PBS and incubated with 10 μM DCFH-DA in PBS for 30 min, at 37ºC in 5% CO_2, protected from light. After incubation, cells were washed with PBS, harvested with trypsin/EDTA and evaluated by flow cytometry (*FACS* AriaII, Becton Dickinson). For MitoSOX™ Red assay, cells grown at 70% confluence were washed with HBSS (with Ca/Mg) and incubated with MitoSOX™ Red (Invitrogen) 5 μM in HBSS (with Ca/Mg) for 20 min at 37 °C in 5% CO_2 protected from light. After incubation, cells were washed with HBSS (with Ca/Mg), harvested with trypsin/EDTA and evaluated by flow cytometry (*FACS* AriaII, Becton Dickinson). Cells without DCFH-DA or MitoSOX™ treatment were used as blank of reaction in each experiment. Ten thousand cells were measured for each experimental condition. Data were analyzed with WinMDI software. Three experiments were performed with triplicates per each experimental condition

Cell proliferation and anchorage independent cell growth

In order to evaluate the cell proliferation, the 3-(4, 5-dimethylthiazol-2-y1)-2, 5-diphenyltetrazolium bromide (MTT, Sigma) growth assay [7] was performed. Briefly 5,000 cells were seeded in 24-well plate and after cultured for 24 and 72 hours, cells were incubated for 2 hours with MTT 200 μg/ml. The formazan formed was dissolved with DMSO and absorbance at 560 nm determined in a plate reader. The experiment was performed three times with quadruplicate measurement per condition.

The anchorage independent cell growth was evaluated trough the soft agar colony formation assay. A bottom layer with 0.5% agar solution in complete medium was performed in 60-mm dishes. Over this layer, 20,000 cells in a 0.35% agar solution in supplemented medium were seeded, cultured 10 days and the number of colonies formed was quantified under microscope. In order to facilitate quantification, 60-mm seeded dishes were divided in similar 16 areas or fields. Results are expressed as average number of colony per field. The experiment was performed three times with triplicates per condition.

ERK and AKT activity

The levels of phosphorylated/total ERK and Akt were determined by western blot as described above and used as a measure of their activities. Anti-p-ERK1/2 and p-Akt antibody 1:1000 were used to visualize the active form of the proteins being relativized to total anti-ERK1/2 1:2000 or Akt 1:1000 antibody respectively (all from Santa Cruz Biotechnology). These primary antibodies were detected using horseradish peroxidase-linked sheep anti-rabbit IgG or donkey anti-mouse IgG, 1:2000 (GE Healthcare). Three independent experiments were performed in duplicate each time.

Cell polarity

Five photographs of cells in subconfluent state were randomly taken with inverted phase-contrast microscope. Length/width ratio (L/W) was calculated using ImageJ software [8]. At least 100 cells were evaluated as follows: length was scored as the Feret's diameter of the cell (longest distance between any two points along the boundary) and width was scored as the secondary axis of the best fit ellipse of the circled cells. Results are presented as the average length/width ratio in arbitrary units (AU). Two independent experiments in triplicates were performed each time.

Classification of different cell types in terms of polarity was performed depending on their apolar, unipolar, bipolar or multipolar appearance (as shown in Figure 2F). An average of 150 cells was evaluated for each condition.

Melanin content

Spectrophotometric assay was performed to evaluate melanin content. Subconfluent cells grown in 100-mm

dishes were washed with PBS and lysed with NaOH 0.1N. Cell suspensions were boiled during 15 min, centrifuged at 15,000 g, 15 min at 4°C, supernatants were recovered and absorbance determined at 400 nm. Protein concentration was determined by Lowry. Results were expressed as absorbance at 400 nm per protein mg. Three independent experiments were done in duplicate each time.

Tyrosinase activity

Activity of cellular tyrosinase was determined spectrophotometrically. Subconfluent cells grown in 100-mm dishes were washed with PBS and lysed in potassium phosphate buffer 0.1 M pH 6.8; Triton X-100 1% plus freeze-thaw cycles. Cell lysates were centrifuged at 10,000 g, 10 min, and protein concentration quantified by Lowry. Reaction mixture in each well contained 40 µg protein of cell lysate, 10 µl 2.5 mM L-DOPA (Sigma) and 0.1 M PBS pH 6.8 (final volume 100 µl). After 1 h incubation at 37°C, absorbance was measured at 492 nm in a microplate reader. Three independent experiments were done in duplicate each time.

TYRP1 protein expression

The levels of TYRP1 protein were assessed by western blot as described above, using rabbit polyclonal anti-TYRP1 antibody 1:1000 (Abcam) and rabbit β-actin antibody 1:1000 (Sigma) as loading control. Both primary antibodies were detected by a horseradish peroxidase-linked sheep anti-rabbit IgG 1:1000 (Amersham, GE Healthcare). Three independent experiments were done in duplicate each time.

Cell response to UV-A radiation

Irradiation source

A bench with two Philips TDL 18W/08 tubes was used as source of UV irradiation. According to the information provided by the manufacturer, more than 95% of the emission from the tubes is at 365 nm. The incident fluence rate under our experimental conditions was measured with a 9811.58 Cole-Parmer Radiometer (Cole-Parmer Instruments Co., Chicago, IL, USA). The UV-A tubes were mounted on aluminum anodized reflectors enhancing the fluence rate on the section to be irradiated. The fluence rate, or the energy per unit area per unit time, was expressed in W/m^2; the total dose, which was calculated based on the fluence rate and the exposure time, was expressed in kJ/m^2 [9].

Irradiation experiment

A total of 5,000 cells were seeded in a 24-well plate and cultured for 24 hours. Cells were washed with PBS and medium without phenol red was used to avoid interference during irradiation. After 24 min irradiation (20 W/m^2 fluence rate and 29 kJ/m^2 total irradiation dose) medium was replaced with fresh one. As control, sham irradiation was simulated by protecting cells from light.

Cell proliferation was evaluated through MTT assay as described above, 24 and 48 hours post irradiation. Results were expressed as the cell proliferation rate post irradiation. Three independent experiments were performed in triplicates.

Assessment of cell migration: Wound healing and transwell assay

Cells were grown in 60-mm dishes until 70% confluence. A scratch in the cell monolayer was performed by using a 200 µl tip. Microphotographs over the whole wound were taken at 0 and 48 hours after healing (Olympus BX51, CCD Camera Olympus DP70). The average width of each wound at both times was determined in arbitrary units using ImageJ software. Three independent experiments were done in triplicates. The extent of cell migration was calculated as the wound width differences between both times.

The transwell migration assay was performed using the CytoSelect™ 24-Well Cell Migration Assay Kit (Cell Biolabs, Inc). Briefly, 300,000 cells were seeded in each transwell and placed in a 24-well plate containing supplemented medium. Cells were cultured during 48 hours and cells that passed through the pores of the membrane were stained and absorbance at 560 nm was quantified following manufacturer's instructions. Three independent experiments were performed in duplicate each time.

Evaluation of cofilin-1, CAP1 and actin polymerization state

Subconfluent cells cultured in 60 mm dishes were fixed in paraformaldehyde 4% in PBS 15 min, washed with PBS, permeabilized with 0.5% Triton X-100 in PBS 15 min and blocked with 5% FBS in PBS 30 min. Cells were incubated with rabbit polyclonal anti-cofilin-1 antibody or rabbit monoclonal anti-CAP1 (both from Abcam) at 4ºC, overnight, and then 1 h with FITC-conjugated anti-rabbit IgG (Sigma). Actin visualization was achieved by rhodamine-phalloidin (Sigma) staining. Finally, cells were counterstained and mounted with 1 µg/ml 4′,6-diamidine-2′-phenylindole (DAPI, Sigma) in an antifade solution. Cells were observed in an Olympus BX51 epifluorescence microscope. Images for each condition were captured by a CCD camera (Olympus DP70). An average of 150 cells was evaluated per experimental condition. To evaluate the levels of cofilin-1 and CAP1 expression, mean fluorescence of each cell was quantified using the ImageJ software. Three independent experiments were performed in triplicates.

Cofilin-1 and CAP1 were also evaluated through western blot (12 and 10% SDS polyacrylamide gels respectively). Rabbit polyclonal anti-cofilin-1 1:2000 or rabbit monoclonal anti-CAP1 1:5000 (both from Abcam) and horseradish peroxidase-linked sheep anti-rabbit IgG (GE Healthcare) were used. As loading control, rabbit β-actin antibody 1:1000 (Sigma) was used. Three independent experiments were performed in duplicate.

For all experiments, protein concentration was measured by Lowry using the DC Protein Assay Reagent (BioRad).

Evaluation of tumor growth and metastasis *in vivo*

Tumor growth was evaluated by determining tumor volume every other day during up to 3 month. The smallest and largest dimensions of tumors were measured with a caliper. Volumes were calculated using the equation: $V = (a \times b^2)/ 2$, where V is the tumor volume, a; the larger dimension and b; the smaller one.

Mice were sacrificed when tumors volume reached around 1000 mm^3 or less and tumors were fixed in formaldehyde 10% in PBS and processed for histopathological examination (H&E staining) and immunohistochemistry.

To study the development of metastasis, nude mice were maintained during 6 month after cells inoculation through tail vein, sacrificed and autopsy of different organs (lungs, brain, liver, spleen and lymph nodes) was conducted. As control of cell viability at the moment of injection, cells were subcutaneously inoculated in the dorsal flank in 2 nude mice per condition. Organs with naked eyes metastasis were fixed and processed for histopathological examination.

Detection of TYRP1, cofilin-1 and CAP1 by immunohistochemistry

For all treatments tissue sections were deparaffinized with xylene and rehydrated through a series of graded alcohols. To detect cofilin-1 antigen retrieval was performed in a water bath for 30 min with sodium citrate buffer (pH 6.0). Endogenous peroxidases were blocked with 5% hydrogen peroxide in methanol and to avoid nonspecific background staining, slides were incubated for 1 h with 1% bovine serum albumin (BSA) (Sigma) in PBS. Rabbit polyclonal anti-cofilin-1 antibody 1:400 (Abcam) was incubated overnight at 4°C. After incubation, HRP-labeled polymer conjugated from Picture-MAX Polymer Kit (Invitrogen) was incubated during 45 min, rinsed, exposed to a diaminobenzidine solution (0.06%) for 5 min and rinsed in running water. After staining with diaminobenzidine solution, sections were dehydrated with alcohol, cleared in xylene and mounted (Entellan, Merck).

For TYRP1 and CAP1 detection, antigen retrieval was equal as for cofilin-1 but the remaining procedure was accomplished by using the Super Sensitive IHC Detection Systems (BioGenex) kit following manufacturer's instructions. Rabbit polyclonal anti-TYRP1 1:100 and rabbit monoclonal anti-CAP1 1:100 antibodies were used (both, Abcam). Next, they were dehydrated with alcohol, cleared in xylene and mounted.

In all cases negative controls were obtained omitting the primary antibody, representing the background staining value in optical density (OD) measurements.

The intensity of each protein IHC reaction was quantitatively measured using an Olympus BX51 microscope coupled to a CCD camera (Olympus DP70) for TYRP1 and CAP1 and a Zeiss Imager AI (2009) microscope coupled to Image-Pro Plus 6.1 software for cofilin-1. Five images were captured for each case on the same day by a single observer. Images were quantified as previously described [10]. Results were expressed as the OD mean values in arbitrary units.

REFERENCES

1. Mruk DD, Cheng CY. Enhanced chemiluminescence (ECL) for routine immunoblotting: An inexpensive alternative to commercially available kits. Spermatogenesis. 2011; 1: 121–122.
2. Aebi H. Catalase *in vitro*. Methods Enzymol. 1984; 105:121–126.
3. Mannervik B. Glutathione peroxidase. Methods Enzymol. 1985; 113:490–495.
4. Wendel A. Glutathione peroxidase. Methods Enzymol. 1981; 77:325–333.
5. Tarpey MM, Wink DA, Grisham MB. Methods for detection of reactive metabolites of oxygen and nitrogen: *in vitro* and *in vivo* considerations. Am J Physiol Regul Integr Comp Physiol. 2004; 286: R431–444.
6. Mukhopadhyay P, Rajesh M, Hasko G, Hawkins BJ, Madesh M, Pacher P. Simultaneous detection of apoptosis and mitochondrial superoxide production in live cells by flow cytometry and confocal microscopy. Nat Protoc. 2007; 2:2295–2301.
7. Liu Y, Peterson DA, Kimura H, Schubert D. Mechanism of cellular 3-(4, 5-dimethylthiazol-2-yl)-2, 5-diphenyltetrazolium bromide (MTT) reduction. J Neurochem. 1997; 69:581–593.
8. Sidani M, Wessels D, Mouneimne G, Ghosh M, Goswami S, Sarmiento C, Wang W, Kuhl S, El-Sibai M, Backer JM, Eddy R, Soll D, Condeelis J. Cofilin determines the migration behavior and turning frequency of metastatic cancer cells. J Cell Biol. 2007; 179:777–791.
9. Pezzoni M, Pizarro RA, Costa CS. Protective role of extracellular catalase (KatA) against UVA radiation in Pseudomonas aeruginosa biofilms. J Photochem Photobiol B. 2014; 131:53–64.
10. Muller CB, de Barros RL, Castro MA, Lopes FM, Meurer RT, Roehe A, Mazzini G, Ulbrich-Kulczynski JM, Dal-Pizzol F, Fernandes MC, Moreira JC, Xavier LL, Klamt F. Validation of cofilin-1 as a biomarker in non-small cell lung cancer: application of quantitative method in a retrospective cohort. J Cancer Res Clin Oncol. 2011; 137:1309–1316.
